# Post-Slaughter Age Classification and Sex Determination in Deboned Beef Using Lipofuscin Autofluorescence and Amelogenin Gene Analysis

**DOI:** 10.3390/vetsci12060593

**Published:** 2025-06-17

**Authors:** Büşra Cumhur, Mustafa Yenal Akkurt, Tuğçe Anteplioğlu, Oğuz Kul, Ufuk Kaya, Bengi Çınar

**Affiliations:** 1 Department of Genetics, Faculty of Veterinary Medicine, Ankara University, Ankara 06110, Türkiye; cmhr.busrra@gmail.com (B.C.); myakkurt@ankara.edu.tr (M.Y.A.); 2 Graduate School of Health Sciences, Ankara University, Ankara 06110, Türkiye; 3 Department of Pathology, Faculty of Veterinary Medicine, Kırıkkale University, Kırıkkale 71450, Türkiye; tugceanteplioglu@kku.edu.tr (T.A.); oguzkul@kku.edu.tr (O.K.); 4 Department of Biostatistics, Faculty of Veterinary Medicine, Hatay Mustafa Kemal University, Hatay 31060, Türkiye; u.kaya@mku.edu.tr

**Keywords:** age determination, *AMELX/AMELY*, beef meat, lipofuscin

## Abstract

Beef meat quality and value are influenced by the breed, sex, and age of slaughtered animals. This study aimed to evaluate lipofuscin pigment autofluorescence as a method for age classification in beef meat samples and to determine the sex of market-obtained meat using PCR-based amelogenin gene amplification. Deboned beef meat samples from *M. longissimus* dorsi and *M. biceps femoris* were collected from 67 slaughtered cows with known age and sex. Additionally, 48 market samples were tested for sex identification and age classification using the same methods. Lipofuscin deposition was first observed at 1.5 years, and autofluorescence analysis effectively distinguished between meat from younger animals (1.5–2.2 years) and older ones (3–13 years), with a statistically significant difference (*p* < 0.001). Lipofuscin levels and excitation intensity increased with age, and no differences were found between the two muscles analyzed. The sex determination results were fully consistent with the records, and 55.2% of animals aged 3 years and older were identified as female. These findings demonstrate the reliability of lipofuscin autofluorescence for binary age determination in beef and support the potential of combining age and sex classification to identify meat derived from older dairy cows in the marketplace.

## Simple Summary

Consumers often expect clear information about the quality of the meat they buy, including the age and sex of the animal it comes from. However, once meat is processed and sold without bones, it becomes difficult to tell whether it came from a young or old animal, or from a male or female. These factors affect meat tenderness, taste, and value. In this study, we tested two scientific methods that can help solve this problem. First, we looked at a natural pigment called lipofuscin, which builds up in animal muscles over time and glows under special light. By examining its pattern in muscle samples, we were able to group meat as coming from either young or older animals. Second, we used a simple genetic test to determine whether the meat came from a male or female. Both methods worked reliably, even on meat purchased from the market. This combined approach could help improve transparency in meat labeling, ensure fair pricing, and protect consumers from misleading information. It may also support regulations in countries where the age or sex of animals matters for religious, cultural, or economic reasons.

## 1. Introduction

Beef meat is an important source of human nutrition and its quality and commercial value have been mainly affected by the sex and age of the slaughtered animal [1,2]. When the cattle carcasses are classified according to their quality, grading is based on the sex, age, and meat maturity of the slaughtered cattle [3,4,5]. The carcass age grading is conducted using ossification and dentition methods in countries such as Canada, the USA (USDA), South Africa, and Australia (AUS-MEAT). However, these examinations cannot be used for age determination in deboned meat and meat products offered for sale. Rising feed costs have led dairy producers to send their animals to slaughterhouses, increasing the presence of meat from older dairy cows in the marketplace [6,7]. Therefore, indicating the sex and approximate age of the animal on product labels, supported by laboratory-based verification when needed, is crucial for transparency. Moreover, female cattle, by virtue of their ability to provide milk, are considered superior in this ethical order to male cattle; beyond the economic implications, in countries like India, where the slaughter of female cattle is prohibited due to religious beliefs, sex determination is essential to ensure accurate labeling for consumers [8,9].

Sex determination can be performed using immunochemical methods based on hormone analysis, including chromatographic techniques and assays such as enzyme-linked immunosorbent assay (ELISA) [8,9,10]. Hormone-based tests are limited by variations in steroid levels due to puberty or hormone treatment and require costly equipment and technical infrastructure, making them unsuitable for routine use [8,9].

DNA-based techniques using specific primers are reliable, economical, and rapid for sex determination. For sex determination, X-linked and Y-linked Zinc Finger Proteins (*ZFX/ZFY*) [11] Testis Specific Protein Y Bound (*TSPY*) [12], a sex-determining region on the Y chromosome (*SRY*) [9,13], a specific segment on the Y chromosome (*BOV97M*), X-linked and Y-linked DEAD-box helicase 3 (*DEAD3X/DEAD3Y*) [8] and steroid sulfatase (*STS*) [14] gene regions, and amelogenin-based sex determination are the most widely used analyses in forensic applications [15]. In cattle, a 63 bp deletion in exon 5 of the amelogenin Y-linked (AMELY), unlike the AMELX, allows sex discrimination by PCR analysis even in low amounts of DNA [16].

Various molecular markers have been explored for age classification. Telomere shortening is one of those markers; however, it varies by tissue and may reflect cell damage rather than replicative senescence alone [17,18]. Although mitochondrial DNA (mtDNA) analysis is promising in humans, its limited sensitivity restricts use in bovine tissues [19]. The signaling common T-cell receptor rearrangement excision circle (sjTREC) method is influenced by the animal’s sex, affecting reliability [20]. DNA methylation (DNAm) analyses have successfully predicted age in humans but lack standardized methods for cattle, though epigenetic clock approaches show potential [17,19,21]. Age prediction using DNA methylation (DNAm) is also influenced by genetics and environmental conditions [22].

Chemical biomarkers and the accumulation of certain compounds in the tissues over time have also been used to determine aging. Lipofuscin (LF), one of the most widely used chemical biomarkers, has been shown to increase with age in many animal species and is not affected by sex or genetics [17,23]. Lipofuscin accumulation has long been recognized as a hallmark of aging in various species, including rodents, fish, amphibians, and marine mammals, where it has been extensively used for estimating biological age due to its progressive, near-linear accumulation in post-mitotic cells [24,25,26]. In aquatic species such as cod, herring, and shrimp, lipofuscin quantification has been applied to otoliths, retinal tissues, and the hepatopancreas to estimate chronological age [27,28]. In mammals, including mice and non-human primates, studies have demonstrated strong correlations between lipofuscin content and chronological age in brain, cardiac, and skeletal muscle tissues [29,30]. Research on livestock remains limited, as meat science has focused on traits like tenderness, marbling, and fat content rather than aging markers. Consequently, lipofuscin—despite its established role in aging studies across species—has not been validated as a practical tool for age classification in beef. Lipofuscin, also called aging pigment, shows a negative correlation with remaining lifespan as it accumulates linearly in cells [25,31]. Lipofuscin, once considered a harmless marker of aging, has recently been reported to be directly associated with the overproduction of reactive oxygen species (ROS); thus, this accumulation is interpreted as detrimental [32].

Lipofuscin accumulation in cattle has been studied in the brain [30,33,34], neural tissue [35], eye [36], spinal cord, adrenal gland, bone, bone marrow, fat, kidney, lung, liver, skeletal muscle, spleen, dorsal root ganglion [37], myocardium [38], heart, and *M. masseter* [33]. Although lipofuscin has been investigated in various bovine tissues, its use as a practical age marker in edible skeletal muscles has not been previously validated. Among the many skeletal muscles in the bovine carcass, *M. longissimus dorsi* and *M. biceps femoris* were selected for analysis due to their large mass, commercial relevance, and histological suitability for lipofuscin scoring. These muscles are frequently sampled in meat quality studies and are easily identifiable across carcasses, making them practical targets for routine screening [1,2]. Furthermore, while current meat grading systems depend on intact carcasses, such classification is not feasible in processed or boneless meat.

Thus, there remains a critical need for simple post-mortem methods to classify the biological age and sex of beef, especially in deboned meat products, for consumer protection and quality assurance. Lipofuscin accumulates progressively in post-mitotic tissues and exhibits strong autofluorescence allowing visual scoring without additional staining, and its stability along with independence from genetic or hormonal factors makes it suitable for age profiling in bovine muscle [23,25,26]. The amelogenin gene, with distinct length polymorphisms on the X and Y chromosomes, enables rapid sex determination even in partially degraded samples [16,39]. Therefore, the aim of this study was to propose and validate a combined approach using lipofuscin autofluorescence scoring for age classification and PCR-based amelogenin genotyping for sex identification directly from deboned, post-mortem meat samples available on the market.

## 2. Materials and Methods

Sample collection: To optimize scoring for lipofuscin analysis, 10 g of *M. longissimus dorsi* and *M. biceps femoris* muscles from 67 beef carcasses (30 males, 37 females) were collected from a slaughterhouse operating under the Ankara Commodity Exchange and fixed in 10% formaldehyde solution. The animals included in the study were selected during two pre-scheduled visits to a slaughterhouse. During each visit, tissue samples were collected from all cattle presented for slaughter on that day, without prior selection, to ensure an unbiased and representative sampling strategy. The collected samples were categorized into three age groups—1.5–2.2 years (n = 30, young adults), 3–6 years (n = 6, middle-aged), and 7–13 years (n = 31, older animals)—in order to represent physiologically distinct stages of bovine development and aging. The first group (1.5–2.2 years) corresponds to animals commonly slaughtered at market weight and represents the majority of cattle processed for meat production. The second group (3–6 years) includes animals likely retained for extended productive purposes, such as breeding or labor, before culling. The third group (7–13 years) consists of aged animals typically removed from the herd due to declining productivity. This age classification reflects prevailing slaughter patterns in the industry, shaped by producer decisions concerning optimal slaughter timing. Moreover, the observed distribution aligns with common herd turnover practices in commercial cattle systems and was influenced by the random, real-time sampling approach employed during slaughterhouse visits. These animals belong to the Holstein Friesian cattle breed and were raised under intensive (indoor) feeding conditions in the vicinity of Ankara, the capital of Türkiye.

To ensure unbiased evaluation, all muscle samples from the slaughterhouse were anonymized and coded by an independent researcher prior to histological processing. The individuals performing lipofuscin scoring were blinded to the age and sex of the animals. Decoding and data grouping were conducted only after all scoring procedures had been completed.

Separate sampling was conducted to allow for blinded evaluation, to validate the lipofuscin scoring method, and to assess the sex distribution of beef products sold in retail markets. The beef samples (n = 48), purchased from markets in different regions of Ankara, were stored in 70% EtOH solution at 4 °C until DNA extraction, which was performed within 3–4 h of collection. For lipofuscin analysis, ten of these samples were fixed in 10% formaldehyde solution within minutes after purchase to preserve autofluorescent structures.

Histochemical Analysis: Tissue samples fixed in 10% formaldehyde for 48 h were trimmed to 2 mm in thickness and washed in running water for 3 h. Routine tissue processing was performed using a Leica TP1020 (Leica Microsystems, Wetzlar, Germany) automatic tissue processor and then the samples were embedded in paraffin. Serial sections of 5 μm thickness were taken on a rotary microtome (Finesse 325, Thermo Electron Corporation, Waltham, MA, USA). The tissue sections were stained with hematoxylin–eosin, covered with a coverslip, and fixed with Entellan. Non-stained sections were deparaffinized, hydrated, covered with aqueous mounting medium, and forwarded for autofluorescence analyses.

Lipofuscin Analysis: Autofluorescence analyses were performed on non-stained tissue sections at 390 nm emission using an upright fluorescence microscope (Leica DM5000 B fluorescence microscope equipped with a Leica DFC425C digital camera, Leica Microsystems, Wetzlar, Germany). All images were captured and evaluated at 10× magnification, with an exposure time standardized at 100 milliseconds to ensure the consistent detection of autofluorescent lipofuscin granules. Higher magnification levels, such as 20×, were avoided during scoring, as they may lead to the misinterpretation of fluorescence intensity or distribution. Both longitudinal and cross sections of the muscle fibers were assessed, and the section orientation offering the most reliable visualization of lipofuscin accumulation was selected for scoring. Specific LF autofluorescent signals were observed as granular and/or homogeneous green emissions alongside the muscle fibers. Nonspecific blue and blue-green fluorescence signals, such as tissue folding, section thickness, and tissue cysts, and autofluorescent signals originating from the perimysium and epimysium exhibiting a sheet-like appearance were not evaluated. To avoid misinterpretation due to tissue autolysis, both fluorescent and brightfield observations were employed. Autolytic muscle tissues typically exhibited high blue to yellowish fluorescence, clearly distinguishable from the green emissions of lipofuscin granules. Under halogen light, autolyzed tissues showed a homogeneous structure and lacked the normal striated pattern of muscle fibers. For each sample, the same regions were evaluated under both light spectra to ensure reliable distinction. The prevalence and severity of lipofuscin emissions were scored according to the Choudhury et al. [40]. In this study, lipofuscin autofluorescence scoring was used to classify meat samples into two age groups: animals younger than 3 years and those 3 years or older. This binary classification framework was chosen in accordance with the Turkish national carcass grading system, which considers 3 years of age a critical threshold in determining meat quality classes. Allred scoring was conducted in five different 10× objective fields. Semiquantitative scoring was performed for the following two criteria:

Autofluorescence area percentage: 0: none, 1: <1%, 2: 1 to 10%, 3: 11 to 33%, 4: 34 to 66%, 5: ≥67%;

Autofluorescence intensity: 0: none, 1: weak, 2; intermediate, 3: strong.

The 2 scores were then added together for a final score with 8 possible values (the Allred score). Note that a score of 1 is not a possible outcome.

DNA Extraction and Quantification: Genomic DNA was extracted using the QuickGene DNA tissue kit S (FUJIFILM Life Science, Cambridge, MA, USA) according to the manufacturer’s protocol. The quality of the DNA was determined using a NanoDrop 2000 (Thermo Scientific, Waltham, MA, USA). DNA degradation was controlled by 1% agarose gel electrophoresis. It was confirmed that the absorbance ratios at 260/280 nm and 260/230 nm were approximately 1.8 and within the range of 2.0–2.2, respectively, indicating high purity of the DNA samples. The DNA extracts were subsequently diluted to a working concentration of 50 ng/µL for downstream applications and stored at −20 °C until use.

PCR Amplification: Primers designed by Ballin and Madsen [39] (F = 5′-CAGCCCCAGICCATCCAGC-3′; R = 5′-GGATGGGGTGCACAGGTGG-3′) were used to amplify two regions, AMELY of and AMELX, with 67 and 130 bases, respectively. Amplifications were performed in 25 μL volume containing 1.5 mM MgCl_2_, 50 ng of DNA, 0.2 μM of each primer, 0.2 μM deoxyribonucleotide triphosphate (dNTPs), 0.5 IU Taq Polymerase (MBI Fermentas), and 10× PCR buffer. Initial denaturation at 94 °C for 4 min was followed by 40 cycles of denaturation at 95 °C for 30 s, annealing at a gradient temperature of 58 °C for 30 s, and extension at 72 °C for 30 s and final extension of 5 min at 72 °C using a C1000 Thermal Cycler (Bio-Rad Laboratories, Hercules, CA, USA). The amplicons was verified using 3% agarose gel electrophoresis stained with SafeView in TBE buffer at 80 V for 30 min. Amplicons were visualized using the Kodak Gel Logic 200 imaging system (Eastman Kodak Company, Rochester, NY, USA).

Statistical Analysis: Descriptive statistics of the pathological scores were calculated and evaluated in terms of parametric test assumptions (normality and homogeneity of variances). Age group differences were analyzed using the Kruskal–Wallis test. Dunn’s multiple comparisons test was used as a post hoc test for variables with statistical significance. All analyses were performed in Stata SE v.15.1 (Statacorp, 2012, LLC, College Station, TX, USA) with *p* < 0.05 considered significant.

## 3. Results

**Figure 1:**
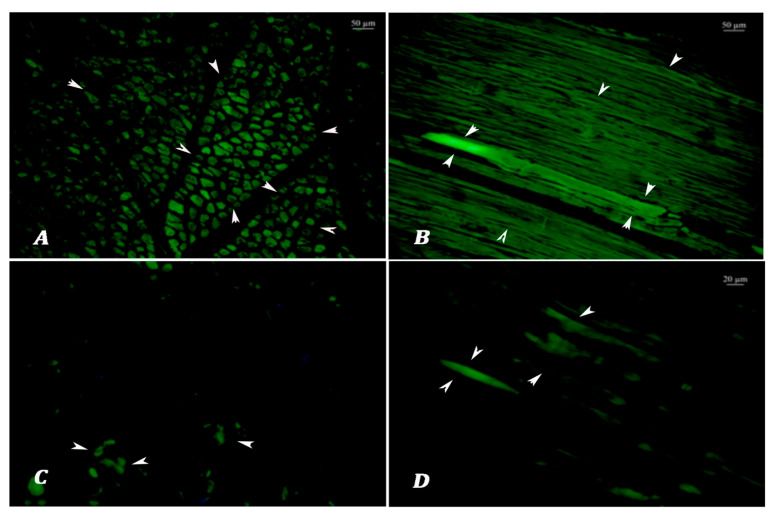
Appearance of lipofuscin autofluorescence (arrowheads) in meat samples from a 6-year-old animal (**A**,**B**) and an 18-month-old animal (**C**,**D**). Green autofluorescence is more prevalent in old animal muscle tissues.

**Figure 2:**
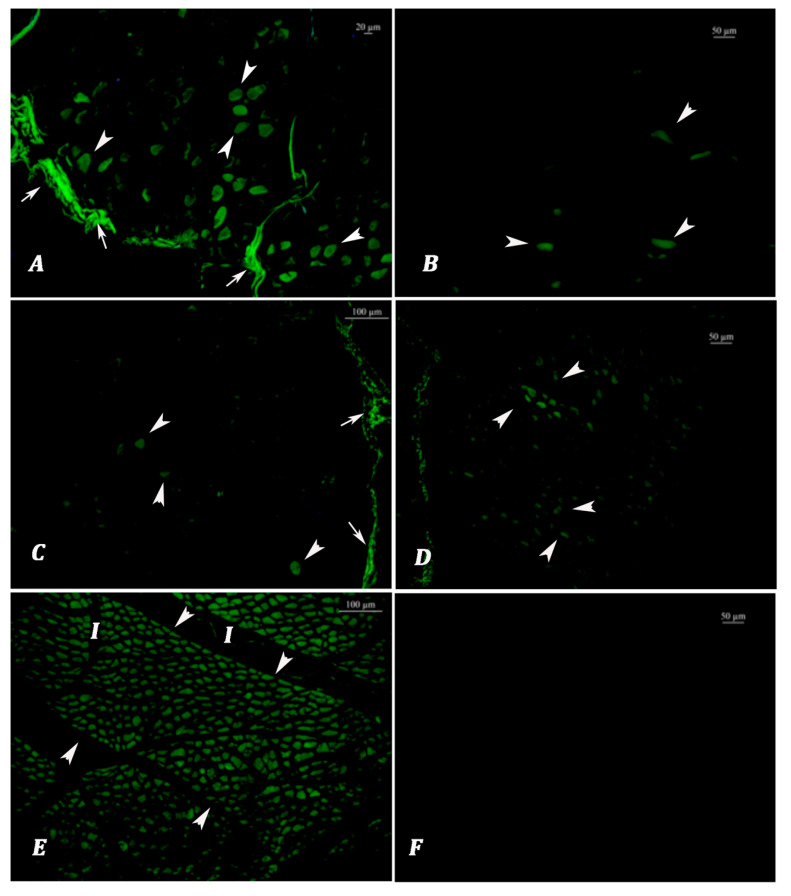
The intensity and dissemination of green autofluorescent emissions from muscle fibers (arrowheads) indicating the presence of lipofuscin accumulation. While very low or no lipofuscin autofluorescence was observed in young animals (**B**,**C**,**F**), dark and/or light green fluorescence emissions were intense in old animals (**A**,**D**,**E**). Perimysium and other connective tissues showed linear and sheet-like non-specific emissions (arrows) at the periphery of muscle groups.

Age Classification According to Lipofuscin Analyses: The lipofuscin depositions of the muscle fibers in cross sections were observed as light to dark green fluorescent emissions (Figure 1 and Figure 2). In the cases that exhibited intense and widespread fluorescent emissions, fibrils were characterized by separated bundles of muscle fibers in cross sections, while linear and spindle-shaped green illuminations were detected in longitudinal sections (Figure 2).

During the blind evaluation of known-age muscle samples, animals aged between 1.5 and 2.2 years were scored in the range of 0–2 due to the absence of or weak fluorescent emissions. In animals aged 3–6 years and older, the change in lipofuscin accumulation increased significantly and showed a difference that increased up to 13 years of age. The lipofuscin autofluorescence scoring for all samples is given in Table 1. The samples collected from the slaughterhouse with known ages were examined, and a statistically significant difference in lipofuscin score was found between the 1.5–2.2-year-old group and the groups ≥3 years old (*p* < 0.001). However, there was no statistically significant difference between the 3–6 and 7–13 age groups (*p* > 0.05). The descriptive statistics are shown in Table 1.

During the evaluation, autolytic and hyalinized muscle fibers were distinguished from true lipofuscin signals based on their distinct fluorescence patterns and corresponding brightfield morphology.

The results of lipofuscin analysis in *M. biceps femoris* and *M. longissimus dorsi* muscle samples collected at the slaughterhouse of animals with known ages due to their records are summarized in Table S1.

According to the lipofuscin analysis method, which was optimized using animals of known age from slaughterhouses, in marketed beef meats, two samples were 3 years old or older, while the others were from animals younger than 3 years old. The results are shown in Table 2.

**Figure 3:**
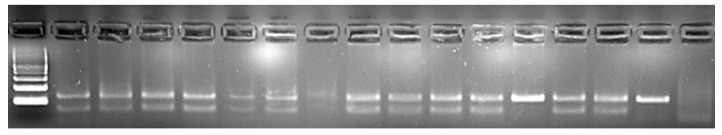
Gel electrophoresis of the amplicons of amelogenin gene regions on X and Y chromosomes. The heterozygous band (130 bp and 67 bp) indicates the male individuals the homozygous band indicates the female individuals. The first lane shows a 100 bp DNA Ladder (Fermantas). Lanes 2–13 show the samples purchased from markets, and lane 14 and lane 15 show the positive controls for males and females, respectively.

Genetic Analysis: Only one of the meat samples (n = 48) collected from the market was determined as female, with the remaining 47 identified as male. Gel electrophoresis images of PCR amplicons of DNA isolated from the meat samples are given in Figure 3.

## 4. Discussion

The accurate classification of the age and sex of beef meat’s origin animal plays a pivotal role in ensuring meat quality, regulatory compliance, and consumer preferences. Since these biological attributes significantly influence carcass grading and market valuation, their identification becomes particularly crucial in processed or deboned meat where traditional anatomical markers are absent. In this context, the present study aimed to establish a practical post-mortem framework for sex identification and age classification using molecular and histochemical tools.

Age classification in cattle is commonly performed using ossification and dentition methods. Ossification techniques evaluate skeletal maturation through radiographic imaging, particularly examining growth plates and epiphyseal fusion in bones such as the clavicle and femur [41]. Dentition methods, on the other hand, rely on the assessment of tooth eruption patterns, wear, and third molar development. These methods offer precise indicators of physiological development, especially when advanced modalities like CT or MRI are used. Nonetheless, environmental and breed-related variations may influence eruption and wear patterns, necessitating population-specific standards for accurate age estimation [41]. These methods rely on intact bones, making them unsuitable for processed or deboned meat. Variability between species, nutrition, and environment also affects ossification timing. Additionally, imaging techniques may involve ionizing radiation, which raises ethical concerns when safer alternatives exist [41,42].

DNA methylation (DNAm) analyses have proven effective for age prediction in humans; however, standardized methodologies for application in cattle are still lacking, despite promising developments in epigenetic clock models [17,19,21]. Hayes et al. [21] showed that it can be used as an epigenetic clock, with mean absolute deviations of 1.4 years and 1.5 years for animals less than 3 years old and 3–10 years old in cattle, respectively. However, DNAm-based age estimation can be influenced by individual genetic background and environmental factors [22]. Lipofuscin (LF), a recognized chemical biomarker of aging, accumulates progressively and almost linearly in post-mitotic tissues over time. Its accumulation occurs independently of sex and genetic makeup and has been shown to negatively correlate with remaining lifespan [23,25,43]. De Biase et al. [30] demonstrated a strong positive correlation between lipofuscin accumulation in neurons and chronological age in aged animals. However, they also reported an absence of detectable lipofuscin in the neuronal tissues of younger animals aged 3–5 years. On the other hand, in our study, lipofuscin granules were observed in muscle samples starting from 1.5 years of age, and the muscle tissue sections examined under the fluorescent microscope demonstrated that animals aged 0–3 years and older than 3 years can be distinguished according to lipofuscin accumulation. As a result of the LF scoring, it is concluded that there is an age-related increase in both muscle groups, *M. longissimus dorsi* and *M. biceps femoris*, which are suitable for lipofuscin examination.

This study focused on assessing lipofuscin accumulation in bovine skeletal muscles as a basis for age group binary classification—using the 3-year threshold defined in meat quality regulations—and aimed to establish a method for classifying age and sex in beef samples obtained from the market. Furthermore, both *M. longissimus dorsi* and *M. biceps femoris* consist predominantly of post-mitotic fibers where lipofuscin granules accumulate progressively with age, enhancing the visibility and consistency of autofluorescence signals [25,26]. Their inclusion in this study was therefore based on both anatomical accessibility and diagnostic value. Based on the results (Table S1), no statistically significant difference was observed between the two muscle types in terms of autofluorescence scores across age groups. This consistency suggests that either muscle can be used for post-mortem age classification in routine applications. However, considering the anatomical accessibility, histological clarity, and lower susceptibility to sectioning artifacts, *M. longissimus dorsi* may offer a slight practical advantage for routine sampling and evaluation. Although lipofuscin has shown high reliability in age classification, its practical use—particularly in bovine species—requires further validation in different tissues and under varying physiological conditions, given its species- and tissue-specific characteristics. Additionally, the low number of animals (n = 6) in the 3–6-year age group may have limited the statistical power to detect significant differences from older animals, and this should be considered when interpreting the findings. In this study, the age groups of slaughterhouse-derived samples were distributed as follows: 1.5–2.2 years (n = 30), 3–6 years (n = 6), and 7–13 years (n = 31). This uneven distribution reflects the natural age demographics of cattle sent for commercial slaughter, where animals are typically slaughtered either at young fattening age or after the end of their productive lifespan as dairy cows [44]. The sample collection was performed randomly and blindly from carcasses available at the slaughterhouse, without prior control over the animals’ age profiles. As such, the formation of equally sized groups was not feasible. Given this distribution and the small sample size in the intermediate group (3–6 years), a non-parametric statistical approach was adopted. Specifically, the Kruskal–Wallis test followed by Dunn’s post hoc test was used, as these methods are robust to violations of normality and unequal group sizes, and are widely recommended for ordinal or non-normally distributed biological data [45]. This approach ensured the statistical validity of group comparisons despite sample imbalance.

Autofluorescent emissions given by autolytic muscle tissues can be distinguished in brightfield examination by the confirmation of hemolyzed erythrocytes in the autolytic tissues. During fluorescent microscope evaluation, microscope illumination can be easily switched from fluorescence to brightfield and does not require the change in slide position to be evaluated. It has been determined that there are other structures (parasites and hyaline degeneration) that show fluorescence in tissue preparations other than lipofuscin. To distinguish these emissions, the appearance of the preparations under fluorescent light and visible light must also be compared. In addition, it was determined that the transverse and longitudinal sections differed in terms of lipofuscin accumulation scores. In this respect, it was concluded that the cross sections to be taken from the tissues are more suitable for evaluating larger muscle masses.

The cross-linking of the cellular components (amine residues) of formaldehyde causes the formation of Schiff base, which is the chemical base responsible for the complex structure between the protein and lipid peroxides that lipofuscin contains and can synthesize lipofuscin-like artificial structures [46]. For the control of the optimized method on the samples purchased from the markets, 10 randomly selected samples from male animals were determined as age classification, of which two belonged to the group of animals 4 years old and above, and eight belonged to the group of animals younger than 3 years old. Two samples of beef meat from market shelves were found to be from animals aged over 4 years old, highlighting the necessity of a grading and classification system to determine meats of different quality and price them accordingly.

It is well established that lipofuscin accumulation occurs not only as a function of aging, but also in response to oxidative stress, mitochondrial dysfunction, metabolic disorders, and degenerative changes in post-mitotic tissues [25,47,48]. In skeletal muscle, conditions such as disuse atrophy, denervation, or chronic inflammation have been associated with increased lipofuscin deposition [29]. Moreover, post-mortem autolytic and oxidative processes may alter the fluorescence properties or increase pigment accumulation [48]. These factors are particularly relevant in market-purchased samples, where the handling history, storage conditions, and animal health status before slaughter remain unknown. Therefore, the potential influence of these confounding variables should be taken into account when interpreting lipofuscin-based age classification.

The results of lipofuscin analysis hold promising practical applications, particularly in the context of forensic meat science and livestock product traceability. If validated for accuracy and reproducibility, this method allows for age estimation directly from meat tissue, even in deboned or processed beef samples where conventional age grading techniques (e.g., ossification or dentition) are no longer applicable. This presents a significant advancement in meat authentication, enabling regulatory agencies, producers, and consumers to verify the age-related labeling of meat products. Additionally, lipofuscin analysis may contribute to the enforcement of age-related slaughter regulations, prevent mislabeling or fraud in meat marketing, and support scientific investigations in cases where the origin or biological age of the carcass is in question. However, further studies are required to develop standardized calibration models that account for breed, muscle type, storage conditions, and oxidative stress levels, all of which may affect lipofuscin deposition rates. The integration of this method into routine meat inspection protocols may enhance transparency and traceability within the beef industry [49].

Sex determination approaches include hormone analysis and DNA-based techniques. Hormone analysis evaluates levels of sex hormones such as testosterone and estrogen, offering a rapid and field-applicable method for inferring sexual maturity [50]. Despite its practicality, hormonal levels are influenced by age, health status, and nutrition, potentially resulting in ambiguous outcomes in immature or diseased animals. In contrast, DNA-based methods assess genetic markers like Y-chromosome sequences, providing highly specific and reliable sex identification. While less affected by environmental factors, these methods require laboratory infrastructure and quality DNA samples, making them more costly and less feasible for routine use in the field [50]. Ultimately, combining these complementary methods and calibrating them to specific populations enhances the reliability of age and sex determination in both forensic and production contexts [41,50,51].

Arslan et al. [11] reported an equal distribution of female and male meat samples (50% each) among 30 beef samples collected from butchers and markets in Kayseri province. In contrast, our study, which analyzed 48 market samples from Ankara using AMELX/AMELY gene region-based PCR, identified only one female sample (2.1%). This striking imbalance is unlikely to be due to sampling bias, as the market sampling was randomized and blinded. Rather, it likely reflects sex-based management and slaughter practices in the Turkish beef industry. Female cattle are generally retained for dairy production and are typically sent to slaughter at older ages, often after the end of their reproductive or productive period. At that point, their carcasses are classified in the third quality category under the national meat grading system [44], which reduces their marketability as prime cuts. As a result, meat from older females is more commonly diverted into ground meat, sausage, or other processed products instead of being sold as retail primal cuts. This discrepancy in sex distribution between slaughterhouse and market samples also raises concerns regarding transparency in meat labeling.

Although all of the market samples in this study yielded PCR-amplifiable DNA, it is important to note that ethanol fixation and extended storage may influence DNA integrity in processed meats. Ethanol is generally considered an effective short-term preservation medium for maintaining DNA quality in soft tissues [52]. Nonetheless, degraded DNA due to suboptimal storage conditions could lead to false-negative results, particularly in low-concentration targets. Furthermore, while deep-frozen samples are likely to retain amplifiable DNA, the application of this method to heat-treated or cooked meat remains uncertain and would require additional validation. Including such sample types in future trials would enhance the practical relevance of the method for food traceability and consumer protection.

Without regulatory requirements for declaring age or sex on consumer products, it becomes difficult to differentiate between high-quality young male meat and downgraded female meat. This highlights the need for traceable, biology-based classification systems supported by molecular verification.

## 5. Conclusions

While the results of this study suggest that lipofuscin scoring has potential as a practical tool for age classification, it is important to note that the method was based on semiquantitative visual scoring without inter-rater validation or objective fluorescence quantification. Although semiquantitative scoring was systematically applied, the lack of numerical assessment for inter-observer variability remains a limitation. Future studies should incorporate standardized inter-rater reliability metrics to enhance reproducibility and analytical consistency.

To improve reproducibility and reduce subjectivity in lipofuscin evaluation, future studies should explore the integration of standardized digital scoring systems and artificial intelligence-based image analysis tools. Advances in fluorescence microscopy and computer vision enable the quantification of autofluorescent signals, which can be leveraged for the automated detection and classification of lipofuscin granules [53,54]. AI-based pipelines, when trained on annotated histological datasets, offer objective high-throughput analysis, minimizing inter-observer variability and enhancing reproducibility. These approaches are increasingly being applied in histopathology and may be adapted to meat inspection systems for large-scale implementation in regulatory or industrial settings [53].

The combined use of lipofuscin scoring and PCR-based sex determination represents a practical post-mortem tool that may contribute to national meat inspection systems. This approach can support the verification of label accuracy regarding age and sex, thereby helping to prevent consumer deception and reduce the risk of fraudulent relabeling. Although current regulations in Türkiye do not mandate the disclosure of biological sex or precise age on meat labels, the integration of molecular methods into meat control programs could improve traceability and transparency. Moreover, in contexts involving religious slaughter or age-dependent carcass grading standards, such tools may offer objective support for certification and regulatory compliance.

In conclusion, the integration of lipofuscin deposition analysis in *M. longissimus dorsi* or *M. biceps femoris* muscles with PCR-based sex identification using AMELX/Y-F and AMELX/Y-R primers may provide a useful strategy for the quality-based classification of beef meat. However, further validation in broader sample sets and under diverse processing conditions is needed to confirm its reliability in routine applications.

## Figures and Tables

**Table 1:** Descriptive statistics of the pathological scoring data obtained from the slaughterhouse. The abbreviations IntScore and AreaPercScore refer to intensity score and area percentage score, respectively.

Parameters	Ages (years)	Mean	Standard Error of Mean	Median	Minimum	Maximum	*p*
IntScore_Total	1.5–2.2	0.55 ^b^	0.22	0.00	0.00	4.00	<0.001
	3–6	3.00 ^a^	0.77	3.00	0.00	5.00	
	7–13	3.77 ^a^	0.28	4.00	0.00	7.00	
AreaPercScore_Total	1.5–2.2	0.73 ^b^	0.21	0.00	0.00	2.00	<0.001
	3–6	3.00 ^a^	0.45	3.00	2.00	5.00	
	7–13	3.60 ^a^	0.45	4.00	0.00	8.00	

a,b: Different superscripts indicate statistically significant differences between groups (*p* < 0.05).

**Table 2:** Lipofuscin scoring and estimated age results of marketed beef meats.

Sample No	Area Percentage (%)	Intensity Score	Total	Classified Age
1	1	1	2	<3
2	2	1	3	>4
3	1	1	2	<3
4	0	0	0	<3
5	2	1	3	≥4
6	0	0	0	<3
7	0	0	0	<3
8	0	0	0	<3
9	0	0	0	<3
10	1	1	2	<3

## Data Availability

The data supporting this study’s findings are available from the corresponding author upon reasonable request.

## Supplementary Materials

The following supporting information can be downloaded at: https://www.mdpi.com/article/10.3390/vetsci12060593/s1, Table S1: Lipofuscin fluorescence scoring of *Musculus biceps femoris* and *Musculus longissimus dorsi* samples getting from the slaughterhouse.
